# HNADOCK: a nucleic acid docking server for modeling RNA/DNA–RNA/DNA 3D complex structures

**DOI:** 10.1093/nar/gkz412

**Published:** 2019-05-22

**Authors:** Jiahua He, Jun Wang, Huanyu Tao, Yi Xiao, Sheng-You Huang

**Affiliations:** Institute of Biophysics, School of Physics, Huazhong University of Science and Technology, Wuhan, Hubei 430074, P.R. China

## Abstract

Interactions between nuclide acids (RNA/DNA) play important roles in many basic cellular activities like transcription regulation, RNA processing, and protein synthesis. Therefore, determining the complex structures between RNAs/DNAs is crucial to understand the molecular mechanism of related RNA/DNA–RNA/DNA interactions. Here, we have presented HNADOCK, a user-friendly web server for nucleic acid (NA)–nucleic acid docking to model the 3D complex structures between two RNAs/DNAs, where both sequence and structure inputs are accepted for RNAs, while only structure inputs are supported for DNAs. HNADOCK server was tested through both unbound structure and sequence inputs on the benchmark of 60 RNA–RNA complexes and compared with the state-of-the-art algorithm SimRNA. For structure input, HNADOCK server achieved a high success rate of 71.7% for top 10 predictions, compared to 58.3% for SimRNA. For sequence input, HNADOCK server also obtained a satisfactory performance and gave a success rate of 83.3% when the bound RNA templates are included or 53.3% when excluding those bound RNA templates. It was also found that inclusion of the inter-RNA base-pairing information from RNA–RNA interaction prediction can significantly improve the docking accuracy, especially for the top prediction. HNADOCK is fast and can normally finish a job in about 10 minutes. The HNADOCK web server is available at http://huanglab.phys.hust.edu.cn/hnadock/.

## INTRODUCTION

Nucleic acids (DNA/RNA) are one of two most important types of biological macromolecules in cells, which are not only transferring genetic information but also involved in various regulatory processes (1,2). Their regulation and function are often realized through interacting with other molecules including nucleic acids (3). For example, RNA–RNA interactions (RRIs) play an important role in many basic cellular activities including transcription regulation, RNA processing and protein synthesis (4,5). As structures determine the functions of molecules, the structural modeling and prediction of such nucleic acid–nucleic acid interactions will be crucial for understanding the molecular mechanism of related biological processes at the atomic level and thus developing therapeutic interventions or drugs targeting the interactions (6–10). Given the high cost and technical difficulties in experimental methods, molecular docking, which computationally predicts the complex structure from individual nucleic acids, is a valuable tool for such structural modeling purpose (11,12). Given two individual molecules, docking samples possible binding modes of one molecule relative to the other. Then, an energy scoring function is used to evaluate and rank the generated binding modes, where the top-scored modes are predicted as the complex structures (13,14).

Despite the importance of nucleic acid–nucleic acid interactions, compared to protein-protein and protein–nucleic acid interactions for which a number of docking algorithms and web servers have been developed to predict their complex structures (14), few approaches have been proposed for the three-dimensional (3D) structural modeling of RNA/DNA–RNA/DNA interactions (11,15), which may be attributed to two reasons. First, compared to proteins, nucleic acids have far fewer experimentally determined structures in the Protein Data Bank (16) as it is much more difficult to determine the structure of an RNA/DNA than a protein. In addition, unlike proteins whose structures can be reliably built through homology modeling (17), RNA/DNA structures are more challenging to be modeled from sequences because sequences are much less conserved than structures when comparing nucleic acids to proteins (15). As structures are critical for the development and validation of docking and scoring methods, the limited number of RNA/DNA structures will significantly limit the development of docking algorithms and scoring functions for RNAs/DNAs. Although some of protein docking programs like HADDOCK (18), GRAMM (19), ZDOCK (20), HEX (21), PatchDock (22), FTDock (23), NPDOCK (24) and HDOCK (25) can conduct RNA/DNA–RNA/DNA docking tasks, their docking accuracies for RNAs/DNAs are limited because scoring functions are not transferable between proteins and RNAs/DNAs (26). Currently, only the web server SimRNAweb is able to model the 3D structure of an RNA–RNA complex based on its intrinsically predicted secondary structures and RNA–RNA interactions or the corresponding information provided by users (27,28). Therefore, a docking web service, which uses an intrinsic scoring function for RNA/DNA–RNA/DNA interactions and is also able to model RNA/DNA–RNA/DNA complex structures from scratch, is pressingly needed.

Meeting the needs, here we have developed a user-friendly web server of our RNA/DNA–RNA/DNA docking algorithm, HNADOCK, in which the putative binding modes are globally sampled through an FFT-based search algorithm (29,30) and evaluated with our intrinsic scoring function DITScoreRR for RNA–RNA interactions (26). Due to the limited number of RNA/DNA structures in the PDB and the difficulty to construct RNA/DNA models, we have also taken advantage of our *ab initio* method 3dRNA for fast RNA 3D structure prediction (31–33). Therefore, HNADOCK server accepts not only structures but also sequences as input, and can automatically integrate the binding site information if provided. The docking process is fully automated and the results are presented to users through an interactive web page and by an email notification if a valid email address is provided.

## MATERIALS AND METHODS

### Workflow of the HNADOCK server

**Figure 1.**
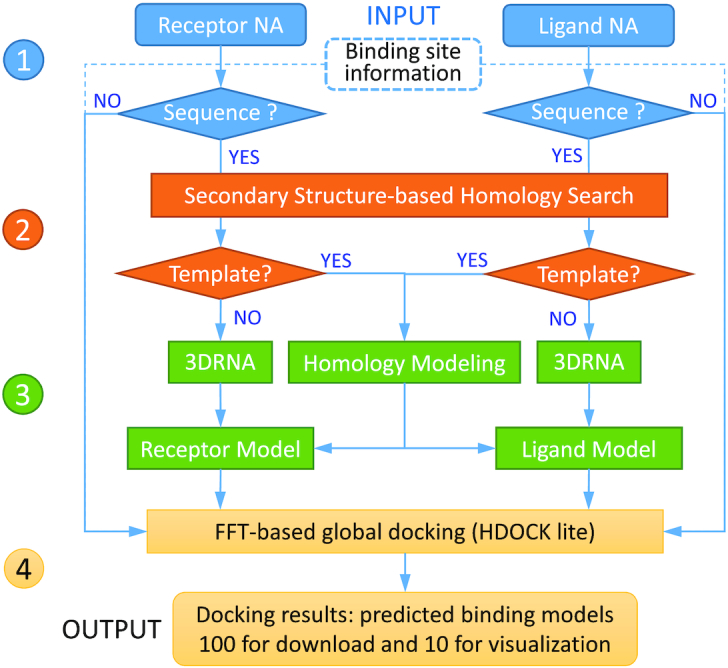
The workflow of HNADOCK server including four stages: (1) data input, (2) homologous RNA search, (3) structural modeling and (4) FFT-based global docking, which are shown in blue, orange, green and yellow, respectively.

HNADOCK server integrates our FFT-based macromolecular docking program HDOCKlite, our intrinsic scoring function DITScoreRR (26) for RNA–RNA interactions, and our *ab initio* RNA tertiary structure prediction algorithm 3dRNA (31). The server also implemented several third-party programs for structural modeling of RNAs, including RNAfold (34), RNAstructure/Fold (35), RNAstructure/MaxExpect (36), RNAstructure/ProbKnot (37) and IPknot (38) for secondary structure prediction, RNAup (39) and RactIP (40) for RNA–RNA interaction prediction, RSmatch (41) for homology search, ModeRNA (42) for comparative modeling of single RNAs, and AMBER (43) for structure refinement. A set of tools developed in our group are used to streamline the docking protocol. The workflow of HNADOCK server is illustrated in Figure 1, which is detailed as follows.

The first step of the docking protocol is to provide two nucleic acid molecules. The server accepts structures for DNAs and both sequences and structures as input for RNAs. Users are also given options to provide the binding site information and choose whether or not to refine the top 10 models.

Then, the server will check the input type of nucleic acid molecules. If the input is a structure, the pipeline will go to the final docking stage. If the input is an RNA sequence, its structure will be built by comparative modeling or *ab initio* RNA structure prediction as follows. That is, a homology search is first conducted against the RNA structure database from the PDB to find possible homologous templates of the RNA sequence by using the secondary structure-based RNA alignment method RADAR/RSmatch (41), where the secondary structure of an RNA is generated by using the RNAfold program of ViennaRNA package (34). If the top hit has an alignment score of >0, the hit will be used as a template of the RNA, and the corresponding 3D model is then built by using the comparative RNA modeling program ModeRNA (42). Otherwise, the 3D structure for the RNA sequence will be constructed by using our *ab initio* RNA 3D structure prediction algorithm 3dRNA (31–33), where the RNA secondary structure can be predicted by RNAfold (34), Fold (35), MaxExpect (36), ProbKnot (37), or IPknot (38).

With the 3D nucleic acid structures modeled by the server or uploaded by users, the workflow enters the last stage, i.e. RNA/DNA–RNA/DNA docking. Here, a hierarchical FFT-based global docking program developed in our group, HDOCKlite (29), is used to sample putative binding modes of one nucleic acid relative to the other. Our scoring function for RNA–RNA interactions, DITScoreRR (26), is used to evaluate and rank the generated binding modes. The docking process will also incorporate the binding site information if users have provided such information at the time of job submission. Specifically, restraints are applied to ensure that the corresponding nucleotides are located at the interface if the binding site on one RNA is provided or within a distance if the constraint information between two RNAs is given during the docking/scoring processes. The docking results are interactively provided to users through a web page and also an email notification if an email address is provided. The top 100 predictions are constructed for download on the result web page, on which users can interactively view the top 10 models through the NGL viewer (44).

### Docking and scoring methods

We have used an FFT-based global docking program, HDOCKlite, to sample the putative binding modes in the HNADOCK server, in which an improved shape-based pairwise scoring function is used for grid-based matching (29). Our scoring function takes into account the contributions not only from its nearest neighboring receptor grids but also from other receptor grids by a form of $\sim e^{-1/r^2}$ in the FFT-based search, where *r* is the distance from a receptor grid point. An angle interval of 15^○^ is used to generate evenly-distributed rotations in the Euler space, and a spacing of 1.2 Å is adopted for the FFT-based matching in the translational search. For each rotation, the top 10 translations with best shape complementarities from the FFT-based search are further optimized by our double-iterative knowledge-based scoring functions for RNA–RNA interactions, DITScoreRR (26). The same scoring function is also used for DNA–DNA and DNA–RNA interactions as our tests showed that scoring functions are transferable between RNAs and DNAs. One binding mode, that corresponds to the best-scored translation, is retained for each rotation. Given the angle interval of 15^○^, there are 4392 evenly distributed rotations in the Euler space (29). Thus, we have a total of 4392 sampled binding modes for a global docking (29). The ranked binding modes are clustered with a ligand root mean square deviation (RMSD) cutoff of 5 Å as used in other docking studies (14), where the RMSD is calculated using the C4′ atoms of RNAs/DNAs (26). Specifically, the first cluster includes those binding modes that are within a ligand RMSD of ≤5 Å from the one with the lowest binding score; the second cluster will include the binding modes that are within a ligand RMSD of ≤5 Å from the one with the lowest binding score after excluding the first cluster of modes; repeat this process until the needed number of clusters are reached or no binding mode is left. For each cluster, the binding mode with the lowest binding score is selected as the representative.

### RNA 3D structure prediction

In the HNADOCK server, we have used our 3dRNA algorithm for RNA 3D structure prediction if an RNA does not have a homologous template. 3dRNA is an automatic and fast RNA tertiary structure prediction method (31). It uses sequence and secondary structure information to build the 3D structures of RNAs from template segments. The workflow for 3dRNA can be roughly described as follows: (i) break the given secondary structure of an RNA into segments; (ii) find the templates of these segments from an RNA segment library, which is pre-built from crystal or NMR structures. If templates are not available, a distance-geometry-based building method will be used to build the segments from scratch. (iii) Construct the final RNA 3D structure by randomly selecting a template for each segment. Therefore, 3dRNA can fast predict multiple structures with given sequence and secondary structure information. Moreover, 3dRNA is able to optimize RNA structures by a Monte Carlo method without breaking the given secondary structure. After that, the k-means clustering algorithm is used to cluster the RNA structure candidates and the 3dRNAscore scoring function (32) is used to choose the appropriate structure among the cluster centers. The accuracy of 3dRNA is highly comparable with other state-of-the-art RNA 3D structure prediction methods (45).

### Input

The required inputs by HNADOCK server are two nucleic acid molecules. Currently, the server only accepts structures as input for DNAs, though it supports both sequences and structures as input for RNAs. For each molecule, the server accepts three types of inputs, two for structures and one for sequences, as follows

- Upload your pdb file in PDB format.
- Provide your pdb file by PDB ID:ChainID (e.g. 1KD5:A).
- Copy and paste your **RNA** sequence in FASTA format.

Only one type of input is needed for each molecule. For structure input, users can upload their own pdb files or provide the PDB: chain ID(s). Since our RNA 3D structure prediction and comparative modeling protocol is designed to build single-chain RNA structures from sequences, users are recommended to upload their own structures if their RNA contain multiple chains.

In addition, users also have an option to provide binding site information in two forms. One is the residue information of the binding site on one molecule. The other is the residue distance constraint at the binding interface between two molecules. The binding site information, if provided, will be used during the docking process as well as the post-docking stage as a filter. A few residues about the binding site is good enough to constrain correct binding modes. An option is also provided for whether or not to refine the top 10 models. Users may also provide an email address for email notification when their job is done, and give a name to their docking job.

### Benchmark

Compared to the prediction of RNA–RNA complex structures, the prediction of DNA–DNA or DNA–RNA structures is less challenging because DNA–DNA or DNA–RNA tends to form a double-helical duplex upon binding, which may be modeled through a purely geometric method. Therefore, we will focus on the case of RNA–RNA complexes in the present test. The test cases used to validate our HNADOCK server are from our nonredundant benchmark for RNA–RNA docking and scoring (46). This benchmark contains 160 diverse RNA–RNA complex structures from the PDB that is clustered with an RNA sequence cutoff of 60% (46). For each case, one or two of the interacting partners are unbound structures. To avoid biases in our evaluation, we have removed those complex structures that were used to train our scoring function DITScoreRR (26), resulting in a total of 60 RNA–RNA complexes in the final benchmark.

### Evaluation criteria

The quality of a predicted RNA/DNA–RNA/DNA binding mode is measured by its interface RMSD (IRMSD) from the native complex structure after optimal superimposition of the predicted and native structures (26). The interface is defined as those residues of the bound structures within 10 Å from the other partner, and the superimposition is based on the C4′ atoms, as used in our previous study (26). A binding mode is defined as a successful prediction or a hit if the interface RMSD between the predicted and native complex structures is <5.0 Å (26). The success rate is defined as the number of cases with at least one correct prediction divided by the total number of cases in the benchmark when a certain number of top predictions are considered.

## RESULTS

### HNADOCK Server

The hardware for HNADOCK server is a Linux server of two Intel(R) Xeon E5-2690 v4 2.60GHz CPUs with 28 cores and 256GB of memory. The software for the web service includes Apache HTTP, PHP and NGL viewer for the docking pipeline and model visualization. The SLURM Workload Manager is used as the job scheduler of HNADOCK server (47). A maximum of 50 jobs can be running at the same time while hundreds of jobs can be queued in the background. The docking process is fast and the average running time for a docking calculation is ∼10 min. The web service does not require registration and is freely available.

After users submit their job, the web interface will be redirected to a web page showing the job ID and running status. The web page is updated every 10 seconds, showing the job status of ‘QUEUED’, ‘RUNNING’, and ‘RESULTS’. The URL to the docking results is something like http://huanglab.phys.hust.edu.cn/hnadock/data/jobid, where ‘jobid’ is a unique job ID. Users can keep the result page alive or bookmark the URL for access to the docking results at a later time. Users will also be notified by email when the job is finished if a valid email address is provided at the time of job submission.

### Output

**Figure 2.**
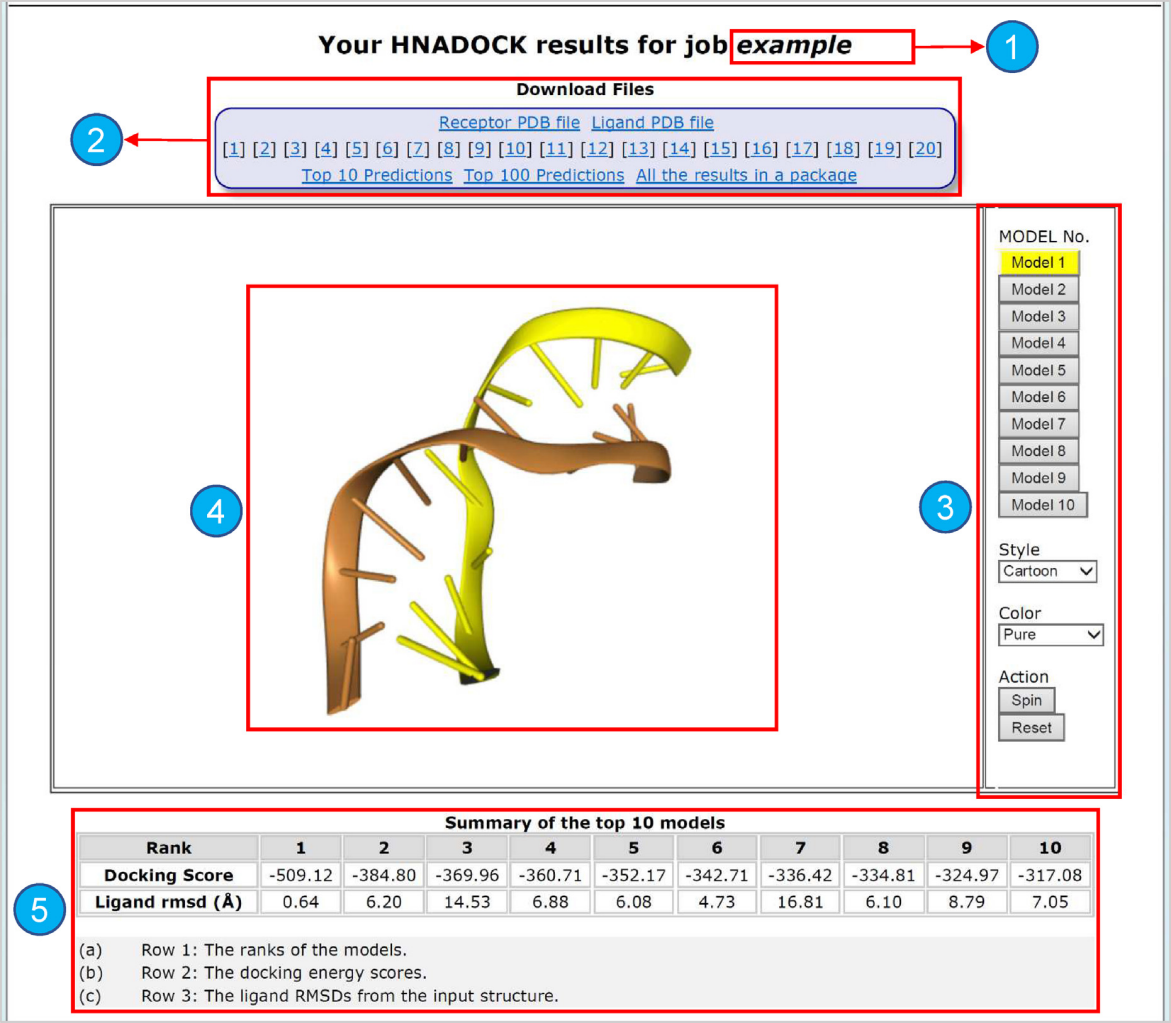
The HNADOCK server result page. At the top of the page is the job name or a unique job ID (1), and the files for download (2). Optional buttons on the right can control NGL to visualize the binding model (3) on the left (4). The docking scores of the top 10 models is shown on the bottom (5).

When a job is finished, the status web page will automatically show the docking results for download and visualization, as shown in Figure 2. The docking results include two types of files for download: the individual pdb files and the docked complex models.

- Receptor and ligand RNA/DNA PDB files uploaded by users or constructed by the server from the FASTA sequence provided by users.
- The server pre-generates the top 100 binding models for each job. Users can download any of the top 20 binding models individually, or choose to download all the top 10 predictions or the top 100 predictions as a package.

Users may also download all the results in a single package that includes the Receptor PDB file, Ligand PDB file, and the top 100 predictions.

As the top 10 binding models are normally deemed as the most important models in macromolecular docking (48), the result page also provides an interactive visualization of the top 10 models using the NGL viewer (44). Users can choose to view any of the top 10 models or all together by different colors, representations, and/or styles.

The result page also gives a summary of the rankings and docking scores for the top 10 complex models, where the score is based on our scoring function for RNA–RNA interactions, DITScoreRR. However, it should be noted that the docking scores here do not reflect the true binding affinities, but a relative ranking of the complex models, as DITScoreRR was not calibrated with experimental binding data (26). It is recommended that users download their docking results as soon as their job is done, as the job results will only be stored on our server for two weeks.

### Performance of the HNADOCK server

As we are developing a web service of our nucleic acid docking for predicting RNA/DNA–RNA/DNA 3D complex structures, we need to ensure that our web server is able to obtain a comparable docking performance to our local docking package. To examine this, we have done two types of validation by providing inputs through the web interface of HNADOCK server. One is to submit the unbound structures to HNADOCK server. The other is to submit the RNA sequences to the server so as to test the ability of HNADOCK server in identifying homologous RNA templates. We then collected the docking results and evaluated the qualities of predicted complex structures. As mentioned in the Benchmark section, we will focus on RNA–RNA docking and thus present the results of HNADOCK server for RNA–RNA complexes only in the present study, though the server also worked well for predicting DNA–DNA and DNA–RNA complex structures per our own test.

**Figure 3.**
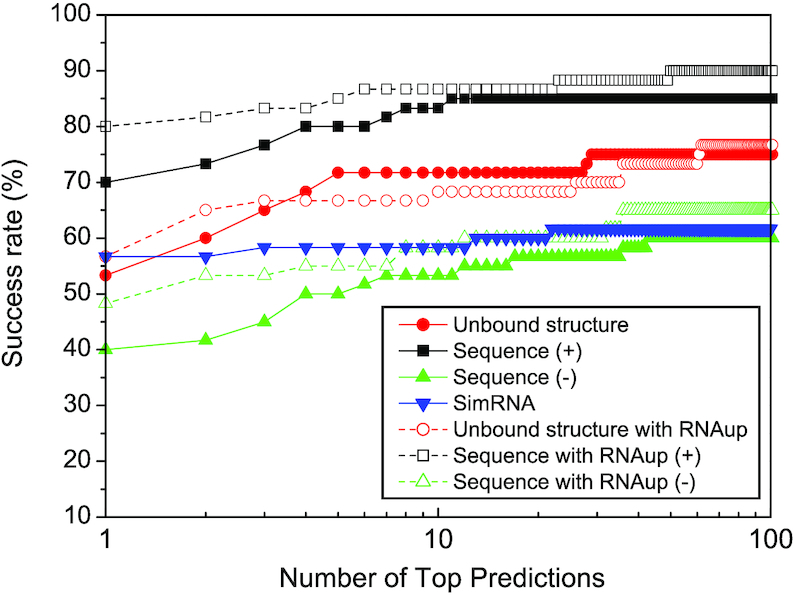
The success rates as a function of the number of top predictions in binding mode predictions by HNADOCK server for unbound structure and sequence inputs with and without using the inter-RNA base-pairing information from RNAup, where the symbol ‘+/−’ stands for modeling single RNA structures with/without including bound RNA templates, respectively. For comparison, the figure also lists the corresponding results of SimRNA.

Figure 3 shows the success rates of our HNADOCK server in binding mode prediction on the benchmark of 60 diverse RNA–RNA complex structures. As a reference, the figure also lists the results of our HNADOCK server with sequence inputs. It can be seen from Figure 3 that HNADOCK server with structure input obtained a high success rate of 53.3% and 71.7% for top 1 and 10 predictions, respectively, which successfully reproduced the docking results of our local docking program shown in our previous study (26). Compared to HNADOCK server with structure input, HNADOCK server with sequence input obtained a significantly higher performance and had a high success rate of 70% and 83.3% for top 1 and 10 predictions, respectively. The better performance for sequence input than structure input can be understood because HNADOCK server with sequence input may have identified some of the bound structures as templates to model RNA 3D structures. To check the robustness of HNADOCK server in structural modeling, we have also tried to remove those RNA structures that have the same sequence as the input from the RNA template database. It can be seen from Figure 3 that HNADOCK server is still able to obtain a satisfactory performance and achieved a success rate of 40% and 53.3% for top 1 and 10 predictions when excluding those bound RNAs from the templates, demonstrating the robustness of HNADOCK server in homologous RNA search (Figure 3).

For comparison, we have also modeled the complex structures of 60 RNA–RNA test cases in our benchmark by running SimRNA (version 3.20) locally, where the SimRNA package was downloaded from the Bujnicki lab and the recommended parameters by the SimRNAweb server were used. Specifically, for each prediction, we carried out eight independent runs of the Replica Exchange Monte Carlo (MC) simulation, each employing 10 replicas. Each run comprised 500 simulation intervals (16 000 steps each) and the lowest energy frame from each interval was recorded. The resulting eight trajectories were combined to yield 40 000 conformations per target (500 conformations from each of the 10 replicas in each of the 8 simulation runs) and the top 1% scored conformations from the set were retrieved and clustered. The clustering threshold was set to 0.1 Å times the sequence length; i.e., 5.0 Å for a sequence of 50 residues. For each cluster, the medoid of the decoys was considered as its representative.

Figure 3 shows the success rate of SimRNA as a function of the number of top predictions. It can be seen from the figure that SimRNA surprisingly achieved a significantly better performance than HNADOCK server for sequence input without including bound RNA templates, and gave a success rate of 56.7% and 58.3% for top 1 and 10 predictions. The good performance of SimRNA is attributed to its two advantages: real flexibility and RNA–RNA constraints, compared to *ab initio* rigid docking. Namely, the MC simulation is able to accommodate the RNA conformational changes upon binding, and the inter-RNA base-pairing information from RNA–RNA interaction prediction can dramatically reduce the sampling space during the simulation, thus enhancing the modeling accuracy. The benefits from RNA–RNA constraints can also be demonstrated by including the similar information into HNADOCK server. As shown in Figure 3, including the inter-RNA base-paring information from RNAup considerably improved the docking performances of HNADOCK and obtained a significantly higher success rate with 80% and 86.7% for sequence input, 56.7% and 68.3% for structure input, and 48.3% and 58.3% for sequence input without including bound RNA templates when the top 1 and 10 predictions were considered, compared to those of *ab initio* docking. It should also be noted that the present SimRNA results are for the cases where the secondary structure or 3D restraints are not supplied by us, but computed by the SimRNA itself. In addition, the secondary structure and 3D restraints used for HNADOCK were predicted by the third-party programs, RNAfold and RNAup. Therefore, the results for both SimRNA and HNADOCK may be further improved with more accurate RNA secondary structure or interaction predictions. The HNADOCK models may also be further optimized by using NA refinement tools like QRNAS (49) or RNAfitme (50) to correct the errors introduced by low resolution modeling and/or rigid docking methods.

### Computational efficiency

**Figure 4.**
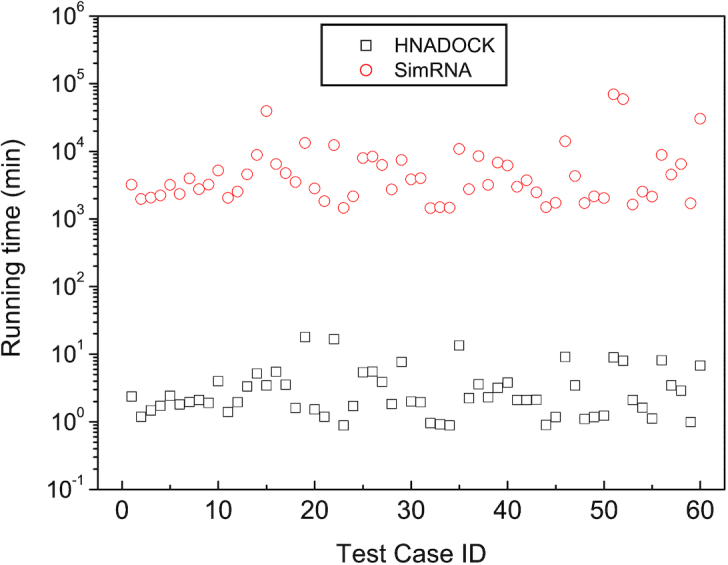
The running times of HNADOCK server and SimRNA for modeling an RNA–RNA complex structure on a single Intel(R) Xeon(R) CPU E5-2690 v4 @ 2.60GHz core over the 60 test cases of the RNA–RNA docking benchmark.

Figure 4 shows the running times of HNADOCK for an RNA–RNA docking job over the 60 test cases in the benchmark. For comparison, the figure also lists the corresponding results of SimRNA for modeling an RNA–RNA complex structure on the same benchmark. It can be seen from the figure that HNADOCK is computationally efficient and can finish a docking job within 10 min for most of the test cases, giving an average of 3.5 min per docking job. In addition, as the first RNA molecular is fixed, the running time of HNADOCK is highly proportional to the length of the second RNA, as expected, and on average consumes about 1.5 min for docking an RNA of 10 nt. As a comparison, SimRNA consumes an average of 7366 min for modeling an RNA–RNA complex structure, which is more than three orders of the time by HNADOCK. The expensive cost of SimRNA can be understood because it runs a lengthy MC simulation to search for the optimal complex structure between two RNAs. It may also explain why the running time of SimRNA is highly correlated with the lengths of both RNAs. On average, SimRNA consumes ∼3000 min for modeling a complex structure between two RNAs of 10 nt.

### Examples

**Figure 5.**
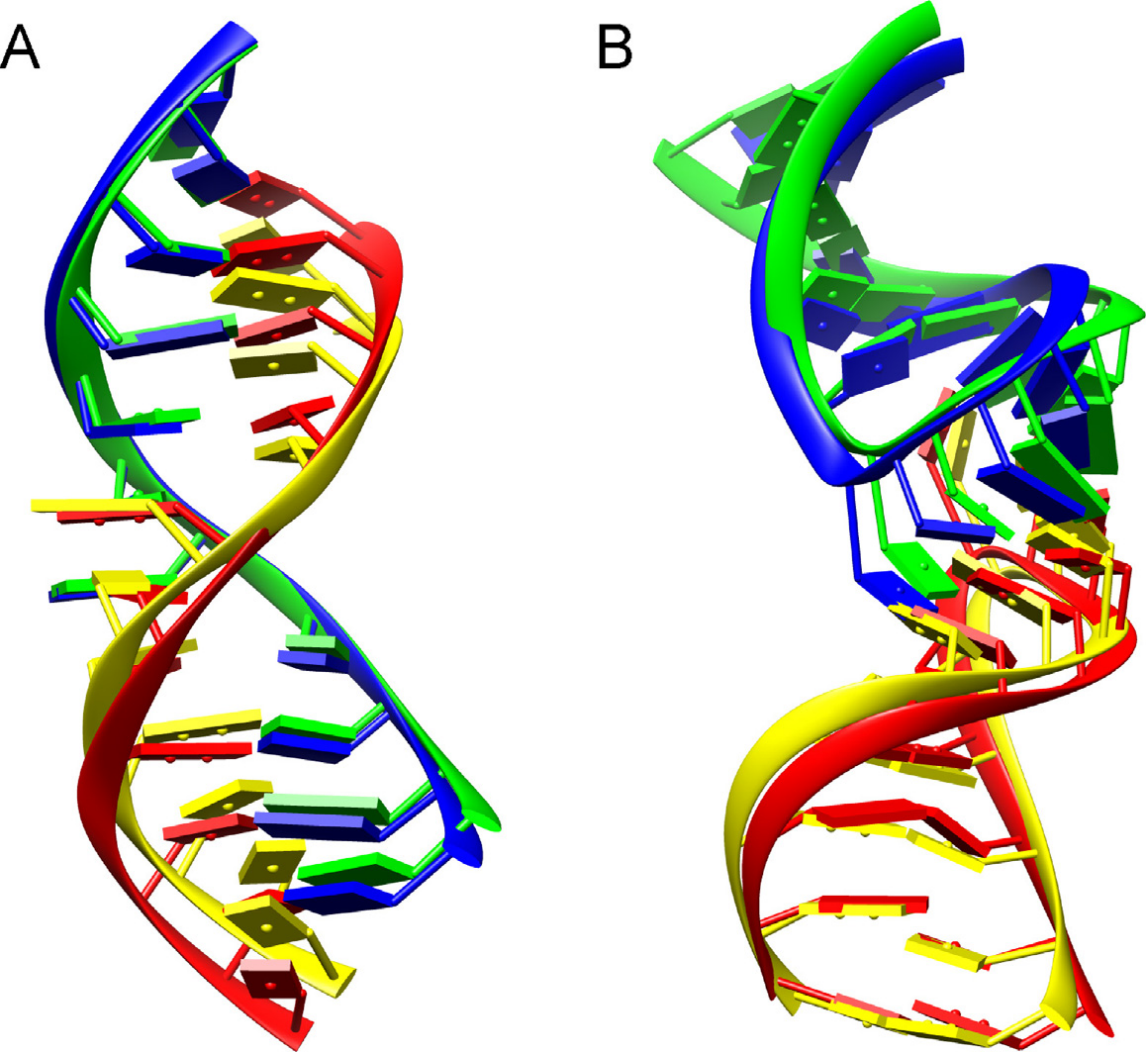
Comparison between the crystal structure (blue and red) and HNADOCK server prediction (green and yellow) for two RNA–RNA docking examples: (**A**) structure input (target code: 1KD5; ranked #1, IRMSD = 1.98 Å); (**B**) sequence input (target code: 1KIS; ranked #4, IRMSD = 2.39 Å),

Figure 5 show two examples of RNA–RNA complex structures built by our HNADOCK server. One is for structure input (Figure 5A), where the two unbound structures of target 1KD5 were submitted to the server as input and no binding site information was provided. It can be seen from Figure 5A that the top predicted binding mode by HNADOCK server successfully reproduced the experimentally determined duplex structure of 1KD5 with an interface RMSD of 1.98 Å. The other is for sequence input (Figure 5B), where the sequences of two RNAs for case 1KIS were submitted to the server and no binding site information was provided. Within the top 10 models predicted by HNADOCK server, the #4 model gives the best consistency with the experimentally determined structure and has an interface RMSD of 2.39 Å (Figure 5B).

## CONCLUSIONS

We have developed HNADOCK, a user-friendly docking web server for modeling the complex structures between two nucleic acids (NAs), where an FFT-based global docking algorithm is used to sample putative binding modes and an intrinsic scoring function for RNA/DNA–RNA/DNA interactions is integrated to calculate the binding energy scores. The server accepts structures for DNAs and both sequences and structures for RNAs, and is also able to include the optional binding site information provided by users. HNADOCK server was extensively tested on the RNA–RNA docking benchmark of 60 diverse complexes and compared with SimRNA. For structure input, HNADOCK server obtained a success rate of 53.3% and 71.7% for top 1 and 10 predictions, compared to 56.7% and 58.3% for SimRNA. For sequence input, HNADOCK server also obtained a satisfactory performance and gave a success rate of 70% and 83.3% when all the RNA templates were used or 40% and 53.3% when excluding those bound RNAs from the templates. It was also demonstrated that including the inter-RNA base-pairing information from RNA–RNA interaction predictions can significantly improve the performances of HNADOCK. On average, HNADOCK consumes about 3.5 min for an RNA–RNA docking job, compared to 7366 min for modeling an RNA–RNA complex structure by SimRNA. These results validated our HNADOCK server as an efficient and reliable docking web server for RNA/DNA–RNA/DNA structural modeling.
